# Unintentional pediatric exposures to household cleaning products: a cross-sectional analysis of the National Poison Data System (2000–2015)

**DOI:** 10.1186/s12995-023-00384-4

**Published:** 2023-08-11

**Authors:** Anthony Pacini, Ben Tsutaoka, Leslie Lai, Timur S. Durrani

**Affiliations:** 1 Division of Occupational and Environmental Medicine, University of California, San Francisco, Box 1369, San Francisco, CA 94143-1369 USA; 2 California Poison Control System, University of California, San Francisco, San Francisco, USA

**Keywords:** Poisoning, Cleaning products, Prevention, Pediatric, Household

## Abstract

**Background:**

Household cleaning products are the second most common cause of unintentional poisoning in children < 6 years old in the United States. The aim of this study is to characterize exposures to household cleaning substances in this age group from data collected from the Nation’s Poison Control Centers.

**Methods:**

This cross-sectional study analyzed all household cleaner calls classified as age < 6 years old made to the American Association of Poison Control Centers between January 1st 2000 and December 31th 2015.

**Results:**

Significant clinical effects or injury was low, making up only 2.6% of cases with a known medical outcome. Alkali-based cleaning products accounted for the third highest frequency of overall exposures and the highest number of all exposure outcomes determined to have a significant clinical effect or injury.

**Conclusions:**

This study demonstrated particular danger of adverse outcomes after exposure to alkali-based cleaning products, specifically alkali-based oven and drain cleaners. Both of which are readily accessible in many households. This study may be a good starting point for further study and poison prevention efforts.

**Supplementary Information:**

The online version contains supplementary material available at 10.1186/s12995-023-00384-4.

## Background

Children comprise a disproportionate number of unintentional exposures reported to Poison Control Centers (PCCs) throughout the United States and nonpharmaceutical household products are the most common substances involved in exploratory ingestions in young children [1–3]. In 2018, the American Association of Poison Control Centers (AAPCC) reports there were approximately 631 poison exposures per 100,000 population reported amongst all age groups, with nearly half (44.2%) of all unintentional poisonings in children < 6 years old. The highest incidence of unintentional poisoning from all categories were in children aged 1 year old, with over 7,000 cases per 100,000 population, followed closely by children aged 2 years old [2]. Household cleaning substances were the second most common exposure in children, making up 10.7% of all pediatric calls aged < 6 years old [2, 4]. The COVID-19 pandemic has only further increased exposures to household cleaning products, with calls to PCCs involving cleaners and disinfectants increasing 20.4% and 16.4% respectively over the period of January to March 2020, when compared to prior years of the same time frame. The largest percentage of exposures coming from children < 6 years old [5]

Unintentional poisonings are also a frequent cause for presentation in emergency departments and engagement in emergency services. A retrospective analysis of Emergency Department (ED) data in 2004 estimated over 85,000 child poisoning incidents in a single year. Of these around 70% were < 2 years old. Cleaning products were the second highest category cited for ED presentations involving unintentional poisoning, making up 13.2% of all cases. In addition, approximately 54.7% of the poisonings involved products already subject to child-resistant packaging requirements under the Poison Prevention Packaging Act [6]. A study analyzing childhood exposure to household cleaning products from 1990 to 2006 estimated 267,269 children were treated in United States EDs for household cleaning product-related injuries [7].

Although in the literature there are specific articles addressing unique pediatric cleaning product exposures, such as to laundry pods [8, 9], it is important to further quantify overall data on unintentional childhood exposures to household cleaning agents. This study provides a descriptive analysis of the AAPCC National Poison Data System (NPDS) database in regard to comparison of medical outcomes per general classes of household cleaners. The aim of this study is to characterize exposures to household cleaning substances in children < 6 years old.

## Methods

The NPDS maintains a database of over 447,000 products, with a significant amount falling into household cleaning products [4]. Data was obtained by formal request to the AAPCC for all cases relating to unintentional cleaning product exposures from 1/1/2000 to 12/31/2015 from the NPDS. Data query used was AAPCC generic category codes for search “CLEANING SUBSTANCES (HOUSEHOLD)”, which resulted in all exposures during the time frame that included 83 unique generic product code numbers/names, see Appendix 1. This analysis of de-identified data was confirmed by self-certification through University of California, San Francisco Committee on Human Research and confirmed by the UC Berkeley Committee for the Protection of Human Subjects to be non-human subjects’ research.

Data obtained from NPDS was filtered to remove exposures with age variable ≥ 6 years old. Exposures with missing data in “Age”, “Gender”, “Generic Code Number” or “Medical Outcome” were excluded. Exposures from the 83 generic product codes were classified by the authors into 14 categories of household cleaning products based on their intended use, major toxic ingredients and modes of action. Specific household cleaning product categories and specific generic product codes were evaluated to delineate which products were associated with more serious medical outcomes. Exposures to multiple household cleaning product categories were not included in comparisons. Definitions for medical outcomes were based on AAPCC definitions [1, 2]. All exposures in this study (total number of calls to PCCs regarding pediatric exposures to household cleaning products) were defined as a population parameter. Therefore, reported outcomes per chemical class are listed as distribution frequencies, are only applicable to the defined population, and are not used to make inferences about a larger population or group exceeding the defined population.

## Results

**Fig. 1:**
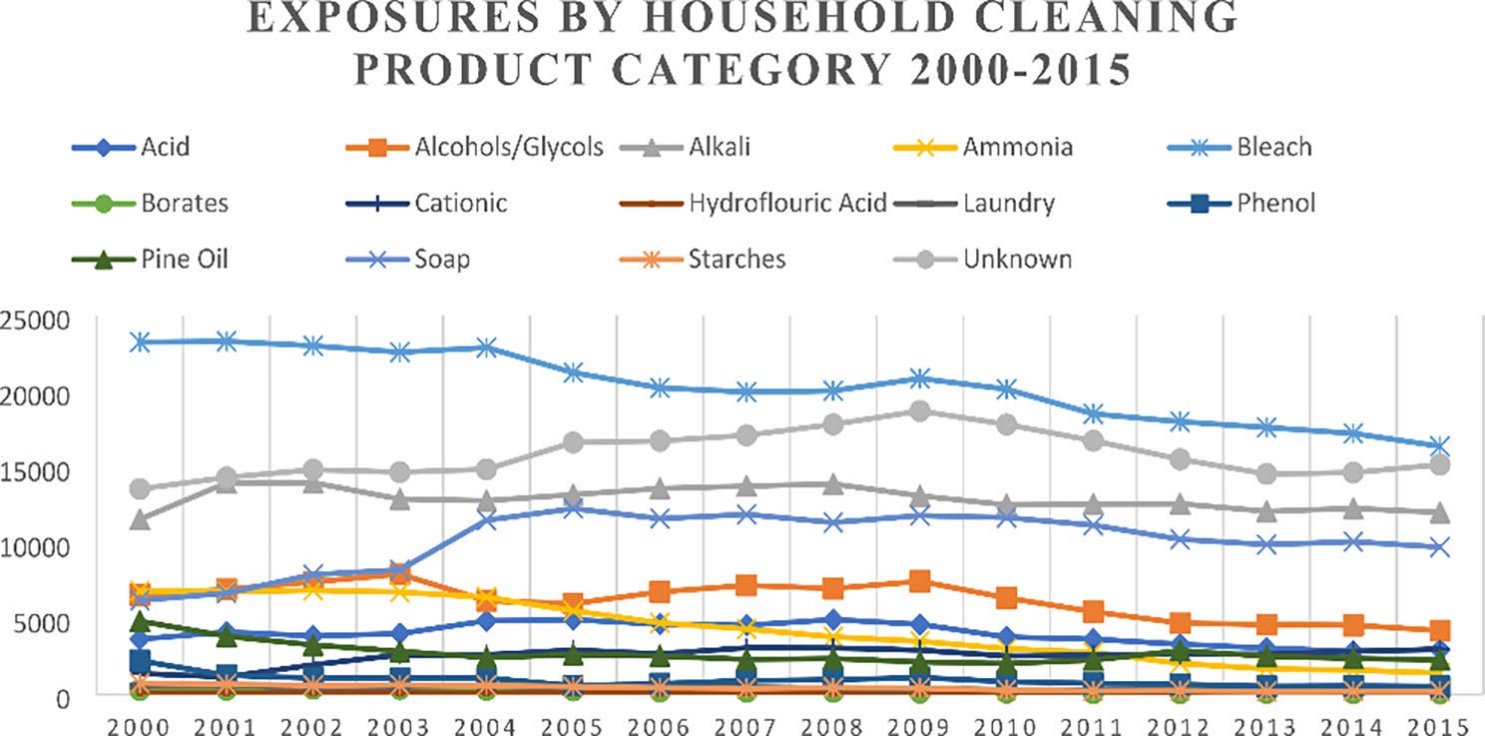
Graphical depiction of household cleaning product category exposure prevalence by year for all pediatric calls 2000–2015 (n = 1,317,970)

There were 1,317,970 pediatric exposures analyzed in the time period. Cases removed for missing data totaled 2,773 (< 0.01% of total). Demographics, exposure site, caller site, management site and route of exposure are presented in Table 1. Total calls per household cleaning product category per year over the study period are represented in Fig. 1. Table 1 about here.

**Table 1:** Demographics for all pediatric exposure calls 2000–2015

Age	n (%)
< 1	116,033 (8.8)
≥ 1 - <2	562,083 (42.6)
≥ 2 - <3	429,694 (32.6)
≥ 3 - <4	127,336 (9.7)
≥ 4 - <5	53,109 (4.0)
≥ 5 - <6	29,715 (2.3)
**Gender**	
Male	778,246 (59.0)
Female	539,724 (41.0)
**Exposure Site**	
Own residence	1,267,961 (96.2)
Other residence	35,359 (2.7)
Public area	4,591 (0.3)
School	4,544 (0.3)
Other	3767 (0.3)
Unknown	886 (0.1)
Health care facility	448 (< 0.1)
Restaurant / Food service	414 (< 0.1)
**Caller Site**	
Own residence	1,113,678 (84.5)
Health care facility	112,879 (8.6)
Other	57,594 (4.4)
Other residence	27,176 (2.1)
Public area	2,894 (0.2)
School	2,361 (0.2)
Unknown	1,269 (0.1)
Restaurant / Food service	119 (< 0.1)
**Management Site**	
Managed on site (non health care facility)	1,145,413 (86.9)
Patient already in or en route to HCF when PCC called	125,204 (9.5)
Patient was referred by PCC to a HCF	35,416 (2.7)
Other	6,921 (0.5)
Unknown	5,016 (0.4)
Route*	
Ingestion	1,136,491 (86.2)
Dermal	259,595 (19.7)
Ocular	143,206 (10.9)
Inhalation/Nasal	24,094 (1.8)
Aspiration	480 (< 0.1)

*Total n exceeds n for exposure cases as a single exposure can have multiple routes listed

HCF = health care facility

PCC = poison control center

Children < 3 years old accounted for over 84.0% of total calls over the time period studied. Exposure site was almost always from a household residence, making up almost 99% of calls. The caller site also demonstrated most calls coming from the residence of the exposed (84.5%). Almost 9% of calls came from a health care facility (HCF). Most of the calls indicated no higher level of care and were managed at home (86.9%), with only a small percentage being referred to a HCF (2.7%) by the PCC, if a patient wasn’t already en route (Table 1). The proportion of exposures by household cleaning product category with the proportion of known medical outcomes, are presented in Table 2. The proportion of known medical outcomes with the criteria meeting or exceeding a moderate outcome was low overall, accounting for 2.6% of total exposures.

**Table 2:** Known medical outcomes per household cleaning product category, n (%)*

Household Cleaning Product Category	Death	Major	Moderate	Minor	No Effect	% ≥ Moderate outcome	Calls Received
Hydrofluoric Acid	3 (0.3)	4 (0.4)	43 (4.6)	349 (37.0)	543 (57.6)	5.3%	1,201 (0.1)
Alkali	0 (0)	261 (0.3)	4,285 (4.3)	34,161 (34.4)	60,582 (61.0)	4.6%	206,538 (15.7)
Acids	0 (0)	53 (0.2)	987 (2.8)	11,834 (34.1)	21,866 (62.9)	3.0%	63,689 (4.8)
Cationic	1 (< 0.1)	15 (0.1)	429 (2.6)	5,756 (34.6)	10,437 (62.7)	2.7%	40,545 (3.1)
Laundry	0 (0)	7 (0.2)	77 (2.3)	1,025 (30.2)	2,280 (67.3)	2.5%	6,608 (0.5)
Bleach	2 (< 0.1)	89 (0.1)	3,507 (2.3)	71,368 (47.1)	76,416 (50.5)	2.4%	325,026 (24.7)
Borates	0 (0)	0 (0)	30 (2.3)	519 (39.7)	759 (58.0)	2.3%	2,885 (0.2)
Soap	4 (< 0.1)	48 (0.1)	1,286 (2.1)	19,093 (30.5)	42,093 (67.3)	2.1%	162,036 (12.3)
Pine Oil	2 (< 0.1)	22 (0.1)	413 (1.9)	6,720 (30.3)	15,044 (67.8)	2.0%	43,463 (3.3)
Unknown	0 (0)	76 (0.1)	1,881 (1.8)	31,488 (30.8)	68,944 (67.3)	1.9%	253,481 (19.2)
Phenol	0 (0)	1 (< 0.1)	105 (1.7)	2,132 (33.9)	4,046 (64.4)	1.7%	14,725 (1.1)
Ammonia	2 (< 0.1)	16 (0.1)	315 (1.2)	8,048 (31.0)	17,621 (67.8)	1.3%	67,920 (5.2)
Alcohols/Glycols	0 (0)	15 (< 0.1)	433 (1.1)	10,977 (28.1)	27,604 (70.7)	1.1%	99,256 (7.5)
Starches	0 (0)	0 (0)	12 (0.6)	417 (22.4)	1,433 (77.0)	0.6%	6,692 (0.5)
Total	14(< 0.1)	607(0.1)	13,803(2.4)	203,887(35.9)	349,668(61.6)	2.5%	1,294,065

* Removed n = 23,905 (1.8%) of cases due to exposures from multiple household cleaning product categories

Alkali based cleaning products were the third most frequent exposure (15.7%) with the highest number of exposures exceeding a moderate medical outcome, n = 4,546 (4.6%) when compared to the other categories. The percentage of moderate or above known medical outcomes in the alkali category was surpassed only by “Hydrofluoric Acid”, which are far less common exposures.

In order to adequately assess the alkali category, it is important to determine what products fall into this class, especially as other chemical classes could be called alkaline when referring to their pH (e.g. bleaches). In this study, the alkali category was defined and delineated into specific generic codes (Table 3). The most common exposure in this group was to all-purpose alkali cleaners including wall, floor, and tile cleaners at 40.1% of all alkali exposures while exposures to miscellaneous alkali-based cleaning agents made up 38.0%. Although alkaline all-purpose agents and miscellaneous agents make up the highest proportion of overall exposure cases, they demonstrated a lower frequency of outcomes exceeding moderate medical outcomes. Conversely, alkali-based oven cleaners and drain cleaners demonstrated the highest proportion of more serious adverse clinical outcomes, 20.9% and 15.4% respectively. However, oven and drain cleaners overall demonstrated a lower frequency of exposure, making up only 3.6% and 3.9% of all alkali-based cleaner exposures respectively. Table 3 about here.

**Table 3:** Alkali known medical outcomes by generic code name, n(%)*

Generic Code Name	Major	Moderate	Minor	No Effect
Drain Cleaners	96 (1.8)	735 (13.6)	1871 (34.7)	2483 (47.9)
Misc Cleaning Agents	57 (0.2)	1239 (3.5)	11,219 (31.7)	22,069 (63.8)
Oven Cleaners	59 (1.2)	965 (19.7)	2493 (50.9)	1245 (26.1)
Rust Removers	0 (0)	0 (0)	5 (31.3)	11 (68.6)
Toilet Bowl Cleaners	5 (< 0.1)	213 (1.4)	3536 (23.1)	10,609 (73.9
Wall/Floor/Tile/All-Purpose Cleaning Agents	44 (0.1)	1133 (2.8)	15,037 (36.5)	24,165 (59.8)

*Death omitted as known medical outcome n = 0

## Discussion

Over the 16-year study period, there were 1,317,970 pediatric exposures to cleaning products reported to the NPDS which met the selection criteria for this study. The majority of exposures occurred in males and those aged ≤ 3 years old. Outcomes exceeding a medical outcome of moderate per AAPCC definition were uncommon, occurring in only 2.6% of all exposure calls. These findings are consistent with previously reported AAPCC data [1, 2]. The overall low risk of household cleaner exposures with the majority of cases being able to be managed safely at home without the need for a higher level of medical care is a continued testament on the importance of the AAPCC and the PCCs they maintain. Critical findings resulting from this project were that a majority of the exposures, 86.9%, required no higher level of care and were managed at home and the alkali category was the third most common exposure to children that resulted in a relatively high rate of medical outcomes exceeding a definition of moderate per AAPCC definition.

A majority of all unintentional poisonings fall on a population where verbal communication and verbal education are absent due to the child’s developmental stage. It is important that effective strategies such as the implementation of engineering and administrative controls to prevent poisoning events are utilized. Emphasizing safe storage and prioritization of access to oven cleaners and drain cleaners during well-child visits may help raise awareness and prevent further significant injury outcomes. Providing free or low-cost locks for lower income families may also be a viable and cost-effective intervention and may be a good focus for a cost/benefit analysis.

There are several limitations of this study. Being cross-sectional in design, with all data coming from an already established data system provided, there is little control over exposure and outcome variables. This can lead to misclassification. When evaluating exposure route, we found “Aspiration” was completely absent for many of the years reported, with an overall prevalence of < 0.1% overall. Whether or not this is true representation of calls or a result of differences in reporting by time, location, individual or re-classification(s) is impossible to determine and one example of how misclassification may exist in this dataset. This study was also unable to account for temporal relationships related to extrinsic and ecological factors outside the data that exist during the time period of the study which may have a confounding effect on relationships presented. NPDS data is representative of the entire population from all national PCCs. This allows evaluation of relationships within this population only. Inference about the true prevalence of unintentional poisoning to household cleaners and their relationships in the United States cannot be made based on this type of study and this data is considered an underestimate of the true prevalence of exposures and likely to have significant misclassification. Data was also self-reported and the majority of cases were unable to be verified.

A particular area of interest not addressed in this study is exposures to cleaning products with child resistant packaging. One of the key agencies involved in regulation of products and child resistant packaging is the U.S. Consumer Product Safety Commission (CPSC). The CPSC was responsible for passing the Poison Prevention Packaging Act (PPPA) in 1970 stating that a household product deemed as hazardous “Must be designed or constructed to be significantly difficult for children under five years of age to open within a reasonable time, and not difficult for normal adults to use properly” [11] Despite this definition, the CPSC stands by a powerful statement that “There is no such thing as child-proof packaging” [11]. There is evidence that child resistant packaging is effective. One analysis of the PPPA found that rates of accidental aspirin ingestion from 1973–1978 decreased 45% following packaging regulations [12]. Alkali based drain cleaners and oven cleaners containing > 10% dry forms of sodium and/or potassium hydroxide or > 2% in other forms, fall under the PPPA. Unfortunately, many household cleaning products are exempt from this act and often the only requirement is a statement of “not child resistant” on the packaging for household products [10].

A final limitation is this study period preceded the COVID-19 pandemic. The pandemic has increased the use of cleaning products, as well as cleaning exposure calls to PCCs^5^, however this study was unable to address any increase pediatric exposures during this time. Further study on the effect of pandemic related cleaning and pediatric exposures should be investigated.

## Conclusions

Exposure to household cleaners is a common cause of unintentional poisoning, especially in the vulnerable population of pediatrics. This study demonstrated particular danger of adverse outcomes in the alkali category specifically alkali-based oven and drain cleaners. Both of which are readily accessible in many households. Alkali-based cleaning products are a demonstrated higher risk chemical class and may be a good starting point for further study and poison prevention efforts.

### Electronic supplementary material

Below is the link to the electronic supplementary material.

Supplementary Material 1

## Data Availability

The data set was obtained by formal request to the AAPCC for all cases relating to unintentional cleaning product exposures from 1/1/2000 to 12/31/2015 from the NPDS.

## List of abbreviations

AAPCC: American Association of Poison Control Centers.
CPSC: Consumer Product Safety Commission.
ED: Emergency Department.
HCF: Health care facility.
NPDS: National Poison Data System.
PCC: Poison Control Center.
PPPA: Poison Prevention Packaging Act.
