# Supplementary material for: Unintentional pediatric exposures to household cleaning products: a cross-sectional analysis of the National Poison Data System (2000–2015)
Source: J Occup Med Toxicol. 2023 Aug 11;18:16. doi: 10.1186/s12995-023-00384-4 (PMC10422824; doi:10.1186/s12995-023-00384-4)
Supplement: Supplementary file 1 — Supplementary Material 1 [file 12995_2023_384_MOESM1_ESM.docx]

Appendix 1: AAPCC CLEANING SUBSTANCES (HOUSEHOLD) Generic Code Name and Number

| **Household Cleaning Product Category** | **Generic Code Name** | **Generic Code Number** |
| --- | --- | --- |
| **Acids** |  |  |
|  | Drain cleaners acid | 0115287 |
|  | Drain cleaners hydrochloric acid | 0201005 |
|  | Drain cleaners sulfuric acid | 0201006 |
|  | Misc cleaning agents acids | 0115282 |
|  | Oven cleaners acids | 0115283 |
|  | Rust removers acids other than hydrofluoric | 0115284 |
|  | Toilet bowl cleaners acids | 0115285 |
|  | Wall/floor/tile/all-purpose cleaning agents acids | 0115286 |
| **Alcohols glycols** |  |  |
|  | Glass cleaners isopropyl alcohol | 0025280 |
|  | Misc cleaning agents ethanol | 0019280 |
|  | Misc cleaning agents glycols | 0051280 |
|  | Misc cleaning agents isopropyl alcohol | 0025281 |
|  | Misc cleaning agents methanol | 0031280 |
|  | Spot remover/dry cleaning agent glycols | 0051281 |
|  | Spot remover/dry cleaning agent isopropyl alcohol | 0025282 |
|  | Wall/floor/tile/all-purpose cleaning agents ethanol | 0019281 |
|  | Wall/floor/tile/all-purpose cleaning agents glycols | 0051282 |
|  | Wall/floor/tile/all-purpose cleaning agents isopropyl alcohol | 0025283 |
|  | Wall/floor/tile/all-purpose cleaning agents methanol | 0031281 |
| **Alkalis** |  |  |
|  | Drain cleaners: alkalis | 0011289 |
|  | Misc cleaning agents alkalis | 0011284 |
|  | Oven cleaners alkalis | 0011285 |
|  | Rust removers alkalis | 0011286 |
|  | Toilet bowl cleaners alkalis | 0011287 |
|  | Wall/floor/tile/all-purpose cleaning agents alkalis | 0011288 |
| **Ammonia** |  |  |
|  | Ammonia excluding cleaning agents – all are multiple ingredient | Multiple |
|  | Ammonia cleaners | 0173280 |
|  | Glass cleaners ammonia containing | 0173281 |
| **Bleach** |  |  |
|  | Bleaches: Hypochlorite (Liquid and Dry) | 0042280 |
|  | Disinfectants: Hypochlorite (Non-Bleach Products) | 0042281 |
|  | Bleaches: Other or Unknown (Household) | 0077282 |
|  | Bleaches: Non-Hypochlorite | 0077280 |
| **Borates (mostly bleaches)** |  |  |
|  | Bleaches borates | 0062280 |
| **Cationics** |  |  |
|  | Industrial cleaners cationics | 0014000 |
|  | Wall/floor/tile/all-purpose cleaning agents cationics | 0014283 |
| **Dishwasher (all are multi)** |  |  |
|  | Automatic dishwasher detergents granules | Multiple |
|  | Automatic dishwasher detergents liquids | Multiple |
|  | Automatic dishwasher rinse agents | Multiple |
|  | Other or unknown type of automatic dishwasher detergent | Multiple |
| **HF** |  |  |
|  | Hydrofluoric acid |  |
|  | Hydrofluoric acid or bifluoride wheel cleaners | 0201007 |
|  | Rust removers hydrofluoric acid | 0118280 |
| **Laundry (mostly spot removers)** |  |  |
|  | Enzyme and/or microbiological laundry additives | Multiple |
|  | Fabric softener/antistatic agents | Multiple |
|  | Laundry bluing and/or brightening agents | Multiple |
|  | Laundry detergent granules | Multiple |
|  | Laundry detergent liquids | Multiple |
|  | Laundry detergent other | Multiple |
|  | Laundry prewash stain removers aerosol | Multiple |
|  | Laundry prewash stain removers liquids | Multiple |
|  | Laundry prewash stain removers liquid surfactants | Multiple |
|  | Laundry prewash stain removers other unknown | Multiple |
|  | Other or unknown laundry additive or miscellaneous product | 0077313 |
|  | Spot remover/dry cleaning agent other hydrocarbon | 0039281 |
|  | Spot remover/dry cleaning agent other halogenated | 0039280 |
|  | Spot remover/dry cleaning agent perchlorethylene | 0170280 |
| **Phenol** |  |  |
|  | Disinfectants phenol | 0040280 |
|  | Misc cleaning agents phenol (excluding disinfectants) | 0040281 |
| **Pine oil** |  |  |
|  | Disinfectants pine oil | 0039282 |
| **Soaps** |  |  |
|  | Anionic or nonionic cleansers | 0013280 |
|  | Anionic or nonionic hand dishwashing detergents | Multiple |
|  | Bath oils and/or bubble baths | Multiple |
|  | Glass cleaners anionics or nonionics | 0013282 |
|  | Industrial cleaners anionics or nonionics | Multiple |
|  | Miscellaneous cleaning agents anionics or nonionics | 0013000 |
|  | Other or unknown type of household hand dishwashing detergent | Multiple |
|  | Oven cleaners detergent type | 0013289 |
|  | Soaps bar, hand, complexion | Multiple |
|  | Spot removers/dry cleaning agents anionics or nonionics | Multiple |
|  | Wall/floor/tile/all-purpose cleaning agents anionics or nonionics | 0013288 |
|  | Anionic or nonionic rust remover | 0013286 |
| **Starches** |  |  |
|  | Starches fabric finishes or sizing | 0036280 |
| **Unknown** |  |  |
|  | Carpet upholstery leather or vinyl cleaners | 0013290 |
|  | Disinfectants other unknown | 0077286 |
|  | Drain cleaners other unknown | 0077314 |
|  | Glass cleaners other unknown | 0077294 |
|  | Misc cleaning agents other unknown | 0077303 |
|  | Other or unknown types of household cleansers | 0077282 |
|  | Oven cleaners other unknown | 0077306 |
|  | Rust removers other unknown | 0077307 |
|  | Spot removers/dry cleaning agents other unknown | 0013287 |
|  | Toilet bowl cleaners other unknown | 0077309 |
|  | Wall/floor/tile/all-purpose cleaning agents other unknown | 0077311 |
